# Triazine‐Trione Thermosets with High Processability for Scaffold Applications in Bone Tissue Engineering

**DOI:** 10.1002/adhm.202504163

**Published:** 2025-11-22

**Authors:** Åshild Johansen, Shuntaro Yamada, Daniel J. Hutchinson, Mohammed A. Yassin, Samih Mohamed‐Ahmed, Cecilie Gjerde, Michael Malkoch, Kamal Mustafa

**Affiliations:** ^1^ Center of Translational Oral Research (TOR) Tissue Engineering Group Department of Clinical Dentistry University of Bergen Bergen Norway; ^2^ School of Engineering Sciences in Chemistry Biotechnology and Health (CBH) Department of Fibre and Polymer Technology Division of Coating Technology KTH Royal Institute of Technology Stockholm Sweden

**Keywords:** bone regeneration, bone tissue engineering, thiol‐ene, thiol‐yne, triazine‐trione

## Abstract

Despite advances in scaffold development, many current biomaterials suffer from limited processability or poor biological performance. To address this, we previously introduced two novel triazine‐trione (TATO)‐based thermosets, T‐ene and T‐yne, as candidates for tissue engineering applications. In the present study, we aim to comprehensively evaluate the initial cellular response of bone marrow mesenchymal stem/stromal cells (BMSC) on TATO materials and their osteogenic potential, with a particular focus on their suitability for bone tissue engineering applications. Both T‐ene and T‐yne demonstrated biocompatibility comparable to polycaprolactone (PCL), as assessed by in vitro cell behavior and the chorioallantoic membrane (CAM) assay. Transcriptomic profiling via RNA sequencing of BMSC cultured on the materials revealed upregulation of mitotic processes in cells on T‐ene compared to T‐yne and PCL. Expression profiling of 92 osteogenic genes after 14 days in osteogenic media showed distinct gene regulation patterns in BMSC on the TATO materials compared to PCL. However, osteogenic differentiation assays, such as Alkaline Phosphatase activity and Alizarin Red R staining, showed no significant differences among the groups. These results suggest that T‐ene and T‐yne are promising, formable, and biocompatible candidates for use in bone tissue engineering applications.

## Graphical abstract

Novel photo‐clickable triazine‐trione thermosets can be shaped and cured under mild conditions, including room and physiological temperatures. These materials are biocompatible and support osteogenic differentiation of bone marrow–derived mesenchymal stem cells on their surface. Their processability and cell‐supportive properties highlight their potential as versatile scaffolding biomaterials for bone tissue engineering and regenerative medicine.

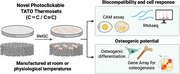

## 1 Introduction

Bone tissue is a dynamic organ with moderate regenerative capacity [1]. However, in cases of critical‐size defects caused by severe trauma, tumor resections, congenital abnormalities or infection, the self‐healing capacity is often limited and insufficient, conventionally requiring surgical intervention with autologous bone grafting. Although autografts are considered the current gold standard, they have several drawbacks, including limited donor tissue availability, risk of donor site morbidity, increased surgical time, and unpredictable resorption post‐transplantation [2, 3, 4]. To overcome these limitations, a novel tissue engineering approach, specifically bone tissue engineering (BTE), has been explored as a promising alternative [5]. BTE strategies typically combine a scaffolding biomaterial with tissue‐forming cells and bioactive molecules [5].

The choice of scaffolding material is critical for the success of BTE, as it needs to meet several key criteria: biocompatibility, osteoconductivity, appropriate mechanical properties, and biodegradability [6]. Scaffolding biomaterials can be broadly categorized into ceramics, metals and natural or synthetic polymers [6, 7]. In particular, synthetic polymers have gained significant attention in the field of tissue engineering due to advantages such as tunable mechanical properties, scalability, and control over architectural features such as shape, pore size, and porosity [8]. Among them, polyesters and polycarbonates are widely studied in tissue engineering. For example, polycaprolactone (PCL), approved by the US Food and Drug Administration (FDA) for use as implantable medical devices, has been exclusively explored as a scaffold for bone regeneration, showing favourable biocompatibility and mechanical strength [9]. PCL, like many thermopolymers, is easily processed, however, its semicrystalline property is a major limitation requiring high temperatures (59°C–61°C) or hazardous organic solvents for shaping and fabrication. This significantly restricts its use in in situ biofabrication or in combination with bioprinting in the presence of living cells [10, 11]. Poly lactic acid (PLA), another synthetic thermopolymer approved by the FDA, also requires high temperatures and/or solvents for shaping and fabrication, and the polymer also possess low mechanical strength and brittleness when used alone [12, 13].

To address this issue, we previously developed an innovative and versatile set of biomaterials based on the 1,3,5‐triazine‐trione (TATO) monomer family. There is a wide spectrum of TATO monomers, several of which are also commercially available and feature different functional groups, yet all share the same central cyclic structure of the TATO ring, resulting in stable and rigid materials [14]. Due to their high functionalization potential, TATO‐based compounds have been investigated for a range of therapeutic uses, including anti‐cancer, anti‐viral, and anti‐inflammatory applications [15, 16]. Trifunctional TATO alkene, alkyne and thiol monomers are known, allowing for the formation of thermosets via high‐energy visible (HEV) light‐induced thiol‐ene coupling (TEC) and thiol‐yne (TYC) coupling chemistry. These click chemistry reactions result in high monomer conversion at ambient temperature and physiological conditions within seconds of HEV exposure and therefore allow for in situ curing of thermosets and composites under surgical conditions. The inclusion of a photo‐initiator results in the generation of radicals upon HEV exposure, which initiate a chemoselective step‐growth polymerization of the thiol and alkene/alkyne monomers [17, 18]. The choice of thiol‐ene or thiol‐yne affects the mechanical properties of the resulting materials with the latter yielding more highly crosslinked stiffer networks [14]. TEC cured TATO‐based thermosets and composites have been explored for use in load‐bearing applications such as fixation patches for bone fractures and dental restorative composites [17, 18, 19]. Specifically, we have designed two photo‐clickable TATO thermosets as scaffolding materials: 1,3,5‐triester‐4‐pentenoic acid‐TATO (TESTATO‐4PA) and 1,3,5‐triester‐4‐pentynoic acid‐TATO (TESTATO‐4PTYA) [14]. These monomers were then combined with a TATO‐based trifunctional thiol, [2‐(3‐mercaptopropionyloxy)ethyl]isocyanurate (TEMPIC), to form mixtures capable of forming thermosets with high crosslink density via HEV induced TEC or TYC chemistry, respectively [14]. The TATO thermosets were successfully fabricated into various forms of scaffolds such as microporous scaffolds with defined pore sizes, thin and elastic sheets, or solid discs at an ambient temperature [14, 20]. The key property distinguishing the TATO thermosets from conventional thermopolymers is the ability to process monomeric mixtures at room and physiological temperatures prior to curing, without the use of hazardous solvents or exposure to temperatures outside the physiological range. This unique feature potentially enables in situ injection and allows fabrication in the presence of living cells. Notably, these thermosets were shown to possess equivalent biocompatibility to PCL and support multi‐lineage differentiation of human bone marrow stem/stromal stem cells (BMSC) [20].

In this study, the TATO‐based thermosets (T‐ene and T‐yne) were further explored biologically using BMSC with a specific focus on cell‐material interaction and their utility for BTE applications. Bulk RNA sequencing was first employed to elucidate early cell‐material interaction, revealing unique cellular responses to the materials in comparison to PCL scaffolds. Fabricating the microporous scaffolds of the TATO‐based thermosets, the biocompatibility and in vitro osteoconductivity was assessed through an ex ovo implantation and osteogenic profiling of BMSC cultured on the scaffolds. The study provides the first comprehensive biological characterization of the novel photo‐clickable TATO scaffolding materials for BTE applications.

## 2 Materials and Methods

### 2.1 Material Preparation

Monomers required for TATO‐thermosets, 1,3,5‐triester‐4 pentenoic acid‐1,3,5‐triazine‐2,4,6(1H,3H,5H)‐trione (TESTATO‐4PA) and 1,3,5‐triester‐4‐pentynoic acid‐1,3,5‐triazine‐2,4,6(1H,3H,5H)‐trione (TESTATO‐4PTYA), were synthesized as previously described [14]. The T‐ene and T‐yne thermoset materials were fabricated by mixing TESTATO‐4PA or TESTATO‐4PTYA with the thiol monomer 1,3,5‐tris[2‐(3‐mercapto propionyloxy)ethyl] isocyanurate (TEMPIC) (Bruno Bock Chemische Fabrik GmbH & Co, Germany) and photoinitiator diphenyl(2,4,6‐trimethylbenzoyl)phosphine oxide (TPO) (415 952; Sigma–Aldrich, USA) and photocuring as previously reported (Table 1) [14]. Medical‐grade polycaprolactone (PCL) (RESOMER C 212; Evonik, Germany) served as a reference material.

**TABLE 1:** Thermoset materials and the concentrations of TPO.

Materials	Alkene	Alkyne	Thiol	Equivalence alkene/alkyne to thiol	TPO [wt.%]
T‐ene	TESTATO‐4PA	—	TEMPIC	1:1	0.25
T‐yne	—	TESTATO‐4PTYA	TEMPIC	0.5:1	0.88

For the fabrication of disc‐shaped scaffolds, T‐ene and T‐yne were cast and photocured in 6‐well plates. Casting PCL in a 6‐well plate required acetone and heat to dissolve PCL, before acetone was left to evaporate. 2 g of polymer per well was used.

For the fabrication of 3D porous scaffolds, a salt leaching technique was employed [21]. PCL (1 g) and T‐ene and T‐yne (2 g) were mixed with acetone and sodium chloride (NaCl) (11 g) with a particle size of 90–600 µm in a 9 cm diameter petri dish. The acetone made the mixture of T‐ene and T‐yne less viscous, enabling the even distribution of NaCl particles. The dishes were left with lids off for the gradual evaporation of acetone. Petri dishes with T‐ene and T‐yne were UV‐cured after acetone evaporation. Constructs with a diameter of 12 mm and a height of 1.2 mm were punched out, unless otherwise specified. Scaffolds were washed thoroughly in distilled water for 96 h under shaking to remove NaCl particles.

Prior to use, the scaffolds were washed in ethanol twice, followed by a wash with DPBS (Dulbecco's Phosphate Buffered Saline, 59300C, Merck, USA), and exposed to UV for 2 h for sterilization.

### 2.2 Micro‐CT Analysis

The 3D porous scaffolds were meticulously scanned using a high‐resolution micro‐CT Skyscan 1172 scanner (SkyScan 1172, Bruker‐MicroCT, Kontich, Belgium). The X‐ray source operated at 40 kV and 200 µA, with no filter applied. For optimal clarity, a medium camera pixel resolution of 2000 x 2000 was utilized. The data sets were then reconstructed using the standardized cone‐beam reconstruction software, NRecon (SkyScan) (*n* = 3).

### 2.3 Cell Culture and Cell Seeding on Scaffolds

Human BMSC were isolated and characterized as described previously after ethical clearance (2020/7199/REK sør‐øst C) [22]. BMSC from three different donors were cultured in a growth medium consisting of α‐minimum essential medium (α‐MEM: 22 571; Gibco, USA) with 10% FBS and 1% penicillin and streptomycin (PS) supplementation in a 5% CO_2_ humidified atmosphere. BMSC from passages 3 to 5 from three different donors were used either separately or pooled for the experiments.

For osteogenic differentiation, BMSC were cultured in an osteogenic medium consisting of α‐MEM with 10% FBS and 1% PS, supplemented with 10 nm dexamethasone (D4902; Sigma, USA), 10 mm beta‐glycerophosphate (G9422; Sigma, USA), and 173 µm L‐ascorbic acid (A8960; Sigma, USA). The medium was changed three times a week.

For the disc scaffolds in 6‐well plates, 150 000 BMSC from three different donors were seeded separately, as well as one sample with pooled cells. For the porous scaffolds in a 48‐well plate, 200 000 pooled BMSC from three donors were seeded.

### 2.4 Seeding Efficiency Assessment

Seeding efficiency was evaluated 4 h after the seeding of 200 000 BMSC onto the porous scaffolds. The scaffolds were transferred to new wells, and both the cells attached to the bottom of the original wells (i.e., escaped cells) and the cells adherent to the scaffolds (i.e., seeded cells) were quantified using PrestoBlue (PrestoBlue HS Cell Viability Reagents: P50201; Thermo Fisher Scientific, USA) in accordance with the manufacturer's protocol (*n* = 5 each). Fluorescence (excitation at 560 nm, emission at 590 nm) was measured in triplicate using a microplate reader (VarioskanTM LUX; VLBL00D0: Thermo Scientific, Finland). The seeding efficiency was calculated as follows:

(1) $$Seeding\;\text{effieciency}=\frac{\left(Seededcells-escapedcells\right)}{Seededcells}\times 100$$

### 2.5 Cytoskeletal Staining and Confocal Microscopy

Porous scaffolds were cultured with BMSC for three days (*n* = 2 each) before being fixed with 4% paraformaldehyde (PFA) for 40 min followed by washing with PBS. Cell attachment and spreading were assessed through cytoskeletal staining by Phalloidin (1:250; A12379, Invitrogen, USA) and nuclear staining by 4′,6‐diamidino‐2‐phenylindole (DAPI, 1:2500; D9542, Sigma–Aldrich, USA).

Samples were imaged using a confocal laser microscope (TCS SP8; Leica, Germany). Z‐projection images were generated using Fiji ImageJ [23].

### 2.6 RNA Extraction and Bulk RNA Sequencing

After two days of culture on the disc scaffolds in the growth medium, BMSC were collected by a cell scraper (*n* = 4).

Total RNA extraction was performed by using a Maxwell 16 Cell LEV simplyRNA Cells Kit (AS1270; Promega, USA) on a Maxwell Instrument (Promega, USA) following the protocol provided by the manufacturer. The extracted RNA was stored at −80°C until further processing. Bulk RNA sequencing (RNAseq) was performed by Novogene Ltd (Cambridge, UK). In short, human mRNA sequencing libraries were prepared from the total RNA using a poly(A) enrichment method. Sequencing was performed on the Illumina PE150 platform, yielding 6 GB of raw data per sample, corresponding to approximately 20 million paired‐end reads.

FASTQ files containing raw sequencing reads were initially processed at the Galaxy (EU‐server) platform [24]. Raw files were uploaded, and the tools Trimmomatic and FastQC were used to ensure sample quality. HISAT2 was used to pair the reads with the reference genome, GRCh38/hg38, and featureCount was used to generate count matrices. After removal of non‐annotated and non‐coding RNA, 27726 genes were identified and used for the subsequent analyses.

EdgeR in Galaxy was used to give a normalized count file and to perform differential expression analysis between groups. Further statistical analysis and data illustration were conducted in R, using the readxl, ggplot2, dplyr, ggrepel, readr, forcats, ComplexHeatmap, circlize, ggvenn packages. Lists of differentially expressed genes (DEGs) were analyzed using the STRING database version 12.0 for protein interactions and Gene Ontology (GO) analysis. Upregulated genes were defined as log2FC < 1 and downregulated genes were defined as log2FC > 1. Files containing enriched biological processes were obtained from STRING and gene ontology graphs showing enriched biological processes were made in R. Gene set enrichment analysis (GSEA) was performed using the software GSEA 4.3.3 with gene set database h.all.v2024.1.Hs.symbols.gmt [25].

### 2.7 Chorioallantoic Membrane Assay

Fertilized eggs (*Gallus gallus domesticus*) were purchased from a local farmer and incubated using an automated egg incubator (Willab AB, Sweden). On embryonic day 3, the eggs were carefully cracked to maintain the chorioallantoic membrane (CAM) and yolk integrity. The egg contents were kept in sterile plastic containers, containing 2 mL PBS with 1% P/S, and incubated in a 37°C humidified environment. On embryonic day 7, the porous scaffolds, with a diameter of 5 mm and a height of 1.2 mm, were placed on top of the CAM. On embryonic day 13, the CAM was dissected and fixed in 4% PFA before further analysis.

Dissected CAM samples were imaged using a stereomicroscope (M205C/MC170HD, Leica, Germany) and further analyzed using an image analysis software (IKOSA CAM Assay Application; KML Vision GmbH, Austria) for quantification. For histological evaluation, the samples were dehydrated through a graded series of ethanol solutions (70%, 80%, 95%, and 100%) for 1 h followed by clearing in xylene for 2 h. Samples were infiltrated with molten paraffin wax at 60°C in two changes, before being embedded in paraffin blocks. Paraffin blocks were trimmed and sectioned into 4–5 µm thick slices using a microtome (RM2155, Leica, Germany). Sections were floated on a water bath at 40°C and then mounted onto glass slides, followed by overnight drying at 50°C. Mounted slides were stained with Hematoxylin and Eosin and imaged using a light microscope (Eclipse 80i, Nikon, Japan).

### 2.8 Osteogenic Gene Array

The porous scaffolds seeded with BMSC were cultured in the osteogenic medium for 14 days and then snap‐frozen until further processing (*n* = 5 each). Total RNA from the collected samples was obtained as previously described following mechanical crushing and sonication. Complementary DNA (cDNA) was synthesized using the High‐Capacity cDNA Reverse Transcription Kit (4368813: Applied Biosystems, USA).

The expression of osteogenic markers was evaluated using a RT‐qPCR array, including 92 osteogenic markers and 4 housekeeping genes (TaqMan Array 96: 4418873; Applied Biosystems, USA). cDNA was mixed with TaqMan Universal Master Mix (4352042; Applied Biosystems, USA) and loaded on the array plates. Amplification was performed using the StepOnePlus Real‐Time PCR System (Applied Biosystems, USA). Genes with cycle threshold (CT) values greater than 35 or below the detectable limit were excluded from the analysis.

The CT values obtained from the gene array for osteogenesis were used to calculate fold change (FC) and log2FC. Further statistical analysis and creation of heatmaps and PCA plots were done in R, using the packages ComplexHeatmap, circlize, dplyr, readxl, FactoMineR, factoextra, and ggplot2. One‐way ANOVA and Tukey's multiple comparisons were performed for the different genes using Prism 10 (Dotmatics, USA). STRING database version 12.0 was used to look at gene interactions.

### 2.9 Alkaline Phosphatase Activity and Alizarin Red S Staining

The porous scaffolds seeded with BMSC were cultured in the osteogenic medium. For measuring alkaline phosphatase activity (ALP), samples were fixed in 4% PFA for 1 min on days 7 and 14 of osteogenic differentiation and stained with BCIP/NBT solution (B5655; Sigma–Aldrich, USA) for 20 min. ALP activity staining was independently repeated twice (*n* = 5 each).

Mineralization was evaluated by Alizarin Red S staining on days 14 and 21 of osteogenic differentiation (*n* = 5 each). Samples were fixed in 4% PFA for 40 min and washed with dH_2_O. Calcium was stained by 2% Alizarin Red solution (A5533; Sigma–Aldrich, USA) in dH_2_O for 30 min, before several washes in dH_2_O on a shaker. The dye was extracted with 100 mm cetylpyridinium chloride overnight to extract the dye. The samples were read in duplicate at 540 nm using the microplate reader, and the analysis was independently performed twice. The data were normalized by dividing the absorbance values by the scaffold surface area, object surface/volume ratio (Figure S1), of each scaffold group, in order to account for differences in available surface area.

### 2.10 Statistical Analysis

All graphs are presented as mean with standard deviation (SD). One‐way ANOVA followed by Tukey's multiple comparisons was used for seeding efficiency, micro‐CT and CAM results, using Prism 10 (Dotmatics, USA), and a *p*‐value < 0.05 was considered statistically significant.

## 3 Results

### 3.1 Fabricated TATO Scaffolds and Characterization

**FIGURE 1:**
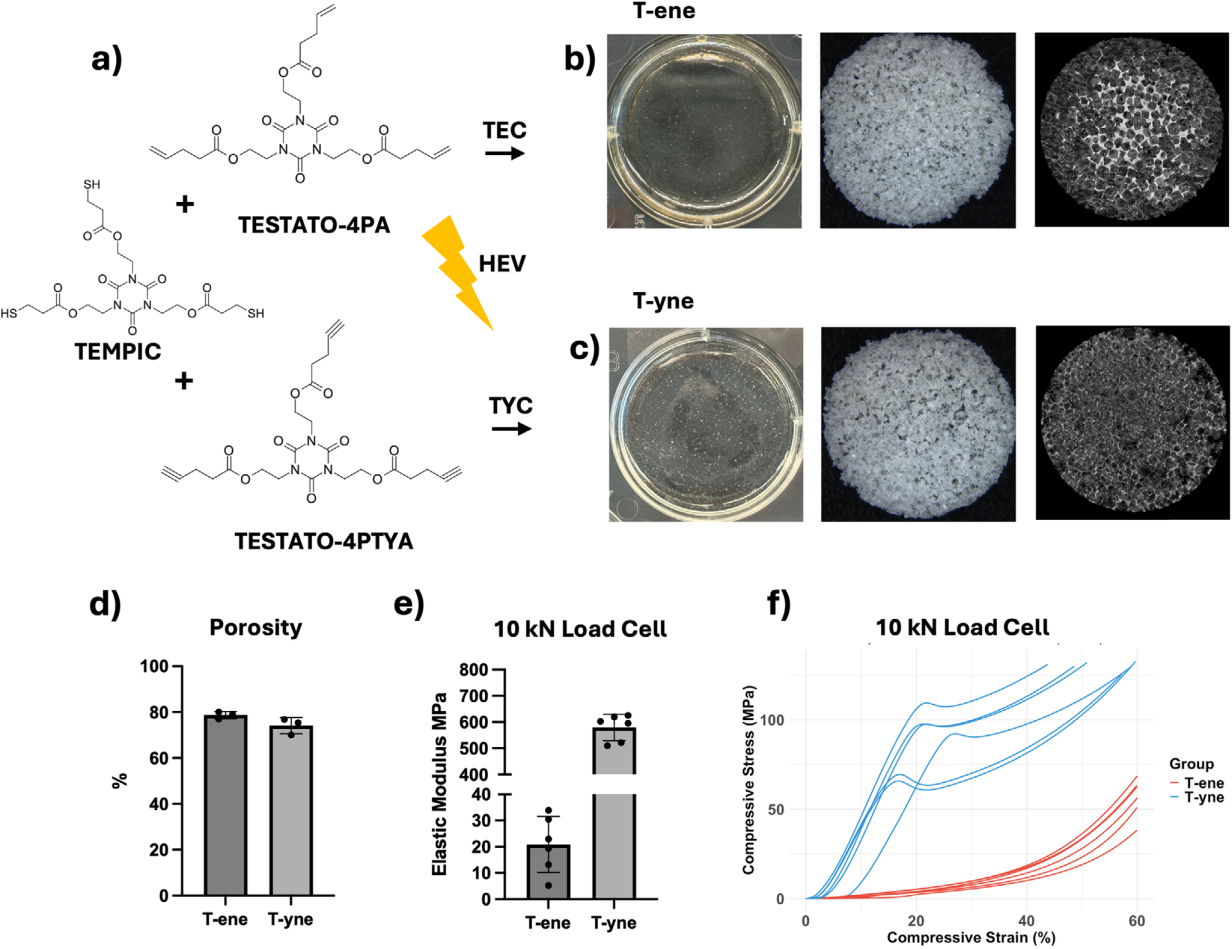
(a) Chemical formula of the monomers; TEMPIC, TESTATO‐4PA, and TESTATO‐4PTYA. (b,c) Wells from a 6‐well plate coated with either T‐ene or T‐yne, macroscopic pictures showing salt‐leached scaffolds of 12 mm in diameter, and micro‐CT images of the salt‐leached scaffolds. (d) Percentage of total porosity obtained from micro‐CT. (e) Elastic modulus of T‐ene and T‐yne. (g)  Stress–Strain curve showing the compressive stress versus compressive strain up to 60% under 10 kN load cell. HEV = high‐energy visible light.

T‐ene and T‐yne showed excellent formability, suitable for fabricating both thin‐film, microporous scaffolds and 3D printed scaffolds using various biofabrication methodologies [20]. In this study, homogeneous precursors of T‐ene and T‐yne were prepared prior to photo‐crosslinking, allowing them to be cast into disk‐shaped scaffolds as well as microporous scaffolds (Figure 1a–c). The designed architectures of microporous scaffold made of T‐ene, T‐yne and PCL were architecturally comparable although some variations in porosity and total surface area were noted (Figure 1d; Figures S1 and S2). T‐ene and T‐yne exhibited markedly different mechanical properties, with T‐ene showing an elastic modulus of 20.84 MPa, in contrast to 579.0 MPa for T‐yne. While the T‐yne specimens reached a distinct failure point, the T‐ene specimens exhibited a continuously increasing modulus without failure (Figure 1e,f).

### 3.2 DEGs on T‐ene and ‐Alkyne Scaffolds Showed Distinct Differences from PCL

**FIGURE 2:**
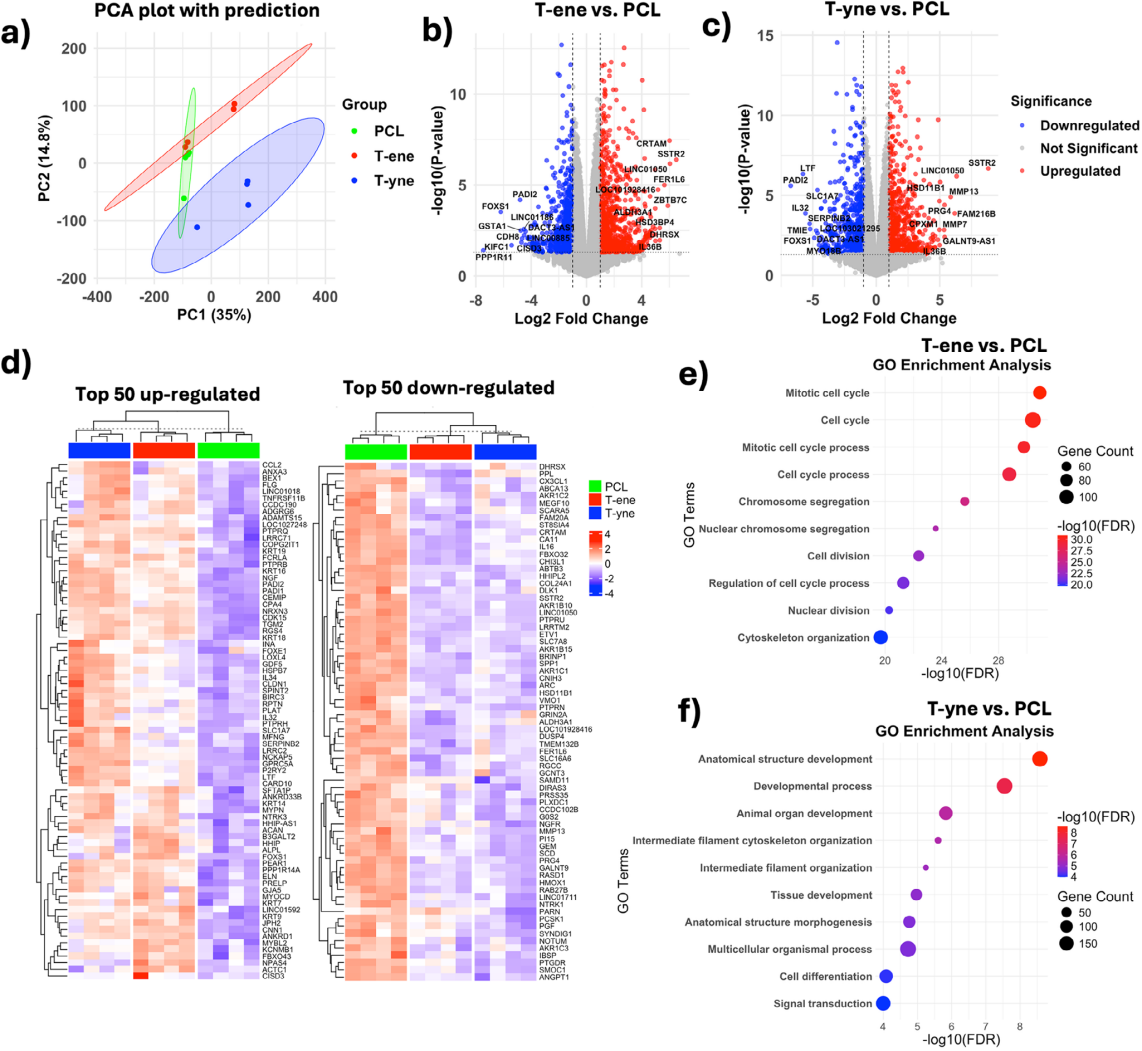
RNA sequencing (RNAseq) results showing (a) Principal component analysis (PCA) with prediction ellipses and (b) Volcano plots of PCL versus T‐ene and (c) PCL versus T‐yne. (d) Heatmaps showing the top 50 up‐regulated genes for T‐ene and T‐yne compared to PCL, and the top 50 down‐regulated genes of T‐ene and T‐yne compared to PCL, with duplicates removed. (e) GO enrichment analysis showing enriched GO terms of T‐ene compared to PCL, and (f) and T‐yne compared to PCL.

BMSC displayed distinct mRNA expression profiles when cultured on T‐ene and T‐yne scaffolds compared to PCL (Figure S3a). Principal component analysis (PCA) showed that the first principal component (PC1) accounted for 34.9% of the total variance, while the second principal component (PC2) explained 14.8% (Figure 2a; Figure S3b). Distinct clustering of the three material groups indicated substantial differences in their feature profiles, with minimal overlap between ellipses, suggesting well‐separated group characteristics. The volcano plots demonstrated clear differences in mRNA expression between the TATO groups and PCL (Figure 2b,c). Compared to BMSC on PCL, 554 genes were upregulated in the T‐ene group, while 504 were upregulated in the T‐yne group. There were 252 upregulated genes shared between the two groups (Figure S3c). Similarly, 723 genes were downregulated in T‐ene and 602 were downregulated in T‐yne, of which 401 genes were common (Figure S3d). Genes universally upregulated in TATO groups included cytoskeletal filaments such as cytoskeletal keratin (KRT7, 9, 14, 16, 18, 19) and cytoskeleton organization factors such as ACTC1, CNN1, ELN, and MYPN (Figure 2d). Functionally, cell differentiation (FDR = 8.20e‐05) and developmental processes (FDR = 2.85e–08) were identified as enriched. Notably, CCL2 was highly upregulated in the TATO groups, suggesting the enhanced immunomodulatory role of BMSC. On the contrary, the TATO groups showed significant downregulation of genes related to oxidoreductase activities (AKR1B10, AKR1B15, AKR1C1, AKR1C2, ALDH3A1, HSD11B1), suggesting altered redox homeostasis. Interestingly, osteogenesis factors such as SPP1, MMP13, IBSP, COL24A1 were also downregulated. Comparing T‐ene and PCL, gene ontology (GO) enrichment analysis indicated that BMSC seeded on T‐ene exhibited a more proliferative phenotype. Biological processes significantly enriched in this group included “mitotic cell cycle,” “cell cycle,” and “mitotic cell cycle process,” suggesting enhanced cellular proliferation in response to the T‐ene substrate (Figure 2e). On the other hand, enriched processes in the T‐yne group were related to differentiation or tissue maturation, represented by “anatomical structure development,” “developmental process,” and “animal organ development ”(Figure 2f).

### 3.3 Enhanced Mitotic Cell Cycle Activity in T‐ene Compared to T‐yne

**FIGURE 3:**
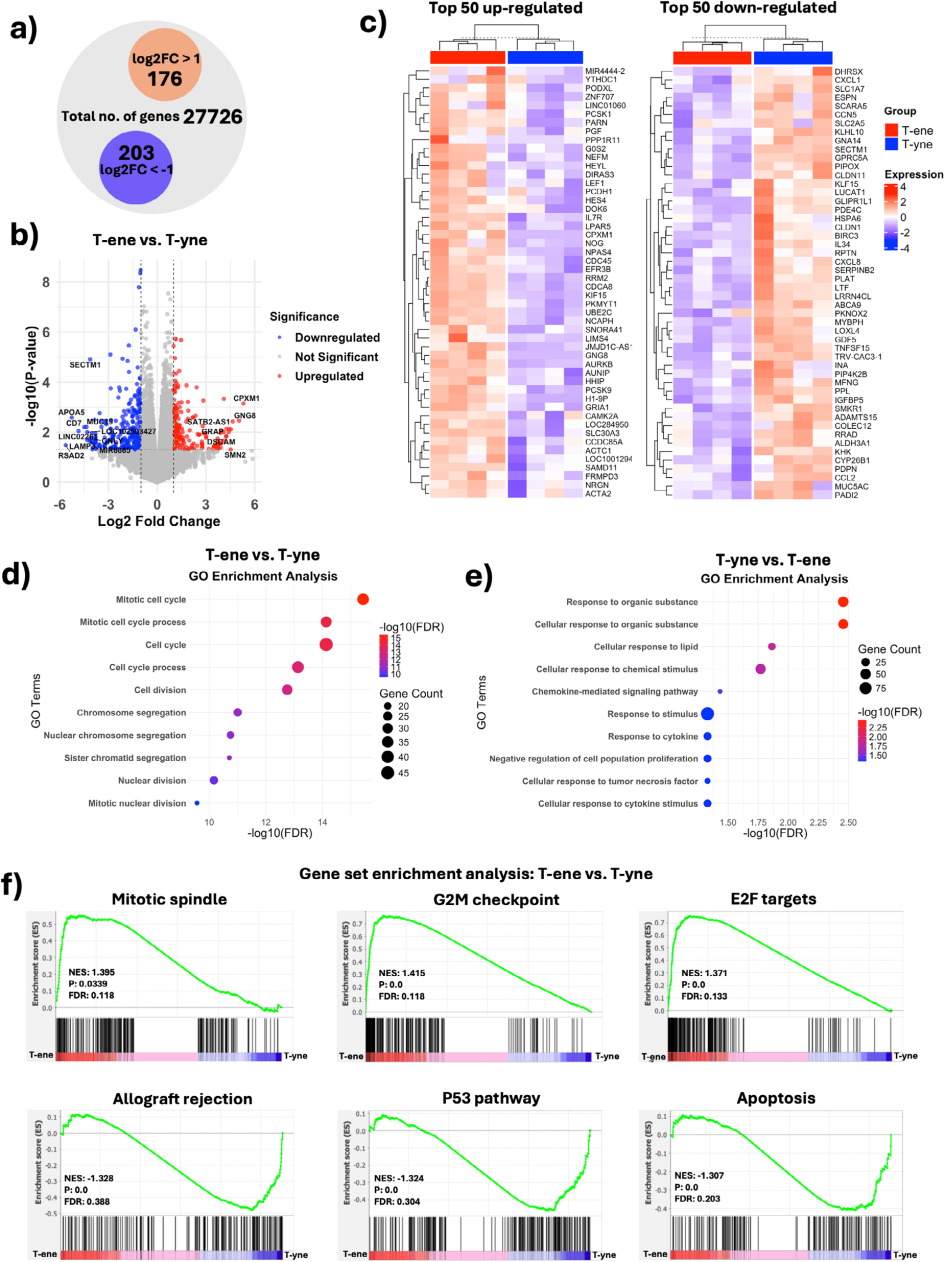
(a) Illustration of number of total genes; 27 726, and the number of up‐regulated genes in T‐ene compared to T‐yne; 176, and the number of down‐regulated genes in T‐ene compared to T‐yne; 203. (b) Volcano plot of T‐ene versus T‐yne. (c) Heatmaps showing the top 50 up‐ and downregulated genes of T‐ene compared to T‐yne. (d) Enriched GO terms of T‐ene compared to T‐yne, and (e) T‐yne compared to T‐ene. (f) Gene set enrichment analysis (GSEA) showing enriched processes in T‐ene; mitotic spindle, G2M checkpoint and E2F targets, and enriched processes in T‐yne; allograft rejection, P53 pathway and apoptosis.

Among the 27726 identified genes, 176 and 203 genes were significantly up‐regulated (log2FC > 1) and downregulated (log2FC < −1), respectively, in BMSC on T‐ene compared to T‐yne (Figure 3a). The volcano plot showed there were significant transcriptional differences between T‐ene and T‐yne, indicating that the different TATO materials had a measurable biological impact on BMSC (Figure 3b). The corresponding heatmaps illustrate the top 50 most upregulated and downregulated genes (Figure 3c). Several of the significantly upregulated genes in the T‐ene group are involved in the regulation of the cell cycle and mitotic division, including “aurora kinase B” (AURKB), “cell division cycle 45” (CDC45), “cell division associated 8” (CDCA8), and “ubiquitin conjugating enzyme E2 C” (UBE2C). Conversely, genes significantly downregulated in the T‐ene group compared to T‐yne included those associated with inflammatory responses and immunomodulation, such as “C‐C motif chemokine ligand 2” (CCL2), “C‐X‐X motif chemokine ligand 1” (CXCL1), “C‐X‐C motif chemokine ligand 8” (CXCL8).

GO enrichment analysis predicted that BMSC seeded on T‐ene enriched biological processes predominantly associated with proliferation‐related functions, including “cell cycle,” “cell division,” and “chromosome segregation” (Figure 3d). In contrast, BMSC on T‐yne exhibited enrichment of biological processes related to environmental sensing and regulation. These included “response to organic substance,” “response to lipid,” “response to chemical stimulus,” “response to cytokine,” and “response to tumor necrosis factor” (Figure 3e). Moreover, the enrichment of “negative regulation of cell population proliferation” in the T‐yne group highlights a divergent cellular response between the two materials. While T‐ene promoted gene expression programs associated with active proliferation, T‐yne appeared to engage more with external signaling and regulatory pathways, including those involved in inflammation and potentially apoptosis, as indicated by enrichment of “cellular response to tumor necrosis factor” [26].

The GSEA reveals statistically significant upregulation of gene sets related to the mitotic spindle, DNA repair, and the G2/M checkpoint in BMSC cultured on T‐ene. In contrast, BMSC, cultured on T‐yne exhibited enrichment in the P53 pathway, allograft rejection and apoptosis (Figure 3f). The P53 pathway is a tumor suppressor pathway and includes regulation of cell death and senescence [27]. Its enrichment, along with pathways related to immune rejection and programmed cell death, indicate that T‐yne may induce early signs of cellular stress or immune activation in BMSC. Notably, additional enriched gene sets for T‐ene included E2F targets, which, along with G2/M checkpoint and mitotic spindle, are closely associated with active cell proliferation and cell cycle progression. In contrast, enrichment of allograft rejection, apoptosis, and the p53 pathway in the T‐yne group highlights a transcriptional signature consistent with negative regulation of proliferation and activation of cell death mechanisms.

### 3.4 Comparable Biocompatibility of T‐ene and T‐yne Scaffolds to PCL

**FIGURE 4:**
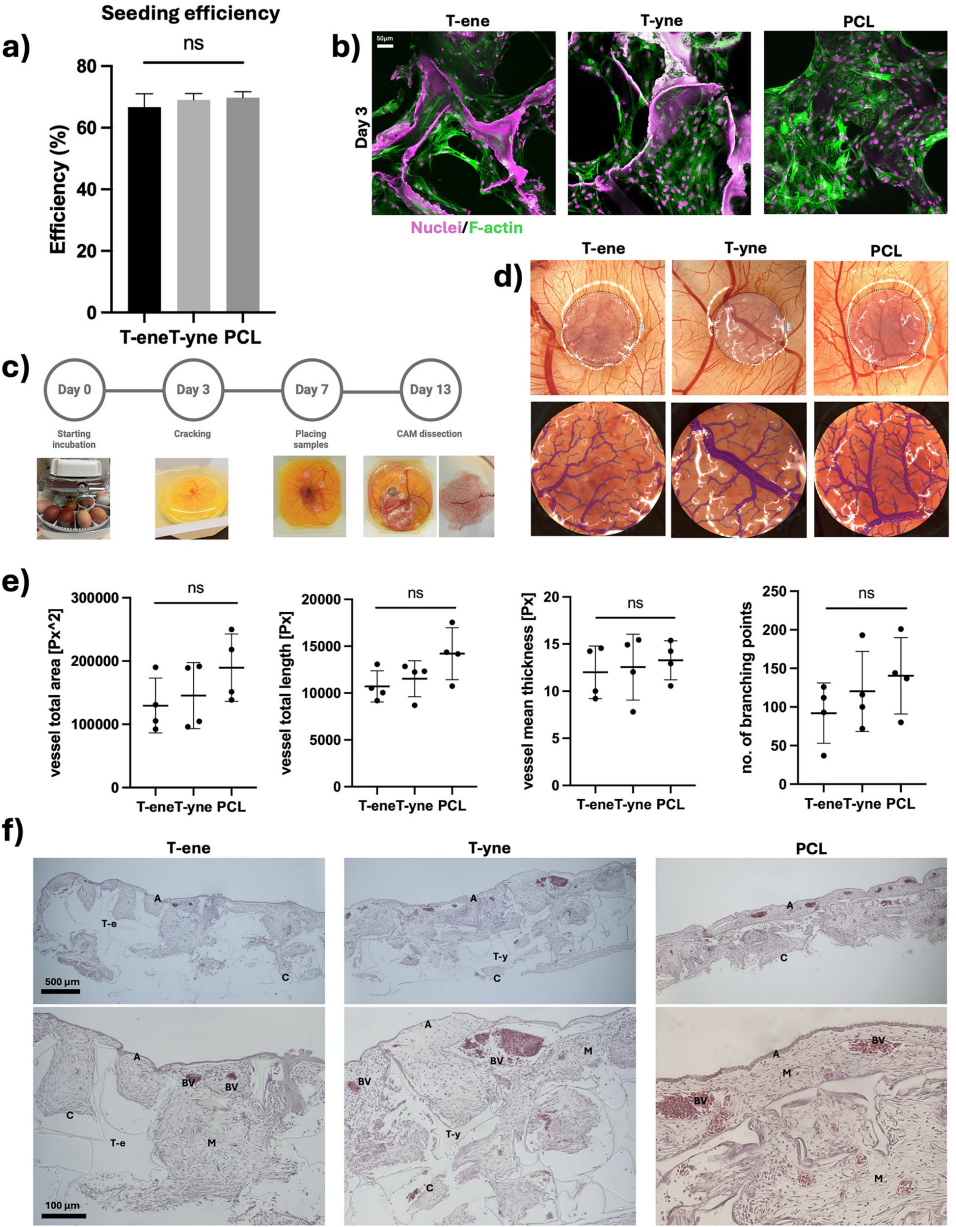
Biocompatibility analysis, showing (a) seeding efficiency measured after 4 h. (b) Attachment and growth of BMSC on the material surface after 3 days of culture. (c) Schematic illustration showing the workflow of the CAM assay (d). Macroscopic image showing the scaffolds integrated in the CAM, the ROI, and the counted vessels. (e) Graphs presenting the total vessel area, the total vessel length, the mean thickness of vessels, and the number of branching points. Histological images of CAM with H&E staining. A = allantoic endoderm, C = chorionic ectoderm, T‐e = T‐ene, T‐y = T‐yne, BV = blood vessel, M = mesenchyme.

When BMSC were seeded onto porous scaffolds composed of T‐ene and T‐yne, a high seeding efficiency of approximately 60%–70% was achieved within the first 4 h of cell seeding, comparable to that observed with PCL scaffolds (Figure 4a). The scaffolds further supported optimal cell attachment, spreading, and elongation over time, indicative of a favorable microenvironment for cellular adaptation and growth. The results were comparable between the thermosets and PCL; however, T‐ene and T‐yne exhibited increased background staining with DAPI, resulting in pink artifacts in the images (Figure 4b).

Biocompatibility was further evaluated by implanting the scaffolds onto the CAM of chicken embryos (Figure 4c). After 5 days of implantation, all scaffolds were integrated into the CAM during the incubation period, suggesting good biocompatibility of the tested materials (Figure 4d) [28]. Notably, neovascularisation was observed within the scaffolds, with T‐ene and T‐yne demonstrating comparable levels of new blood vessel formation to PCL scaffolds, as assessed by total vessel area, total vessel length, average vessel thickness, and the number of branching points. (Figure 4e). Histologically, all the tested materials were encapsulated by the mesenchymal tissue within the intermediate mesodermal layer of CAM (Figure 4f). These results indicated that T‐ene and T‐yne scaffolds exhibited comparable biocompatibility to PCL scaffolds.

### 3.5 Altered Osteogenic Marker Expression Profile in BMSC on T‐ene and T‐yne Scaffolds

Osteogenic profiles of BMSC were tested by assessing the expression of 92 osteogenic markers (Figure S4a). The PCA plot, where the PC1 account for 33.7% of the total variance, while the PC2 accounts for 16.6%, revealed distinct expression patterns among the tested groups, with T‐ene exhibiting the greatest heterogeneity. While some overlap was observed between T‐ene and T‐yne, both TATO‐based groups remained separated from PCL (Figure S4b).

**FIGURE 5:**
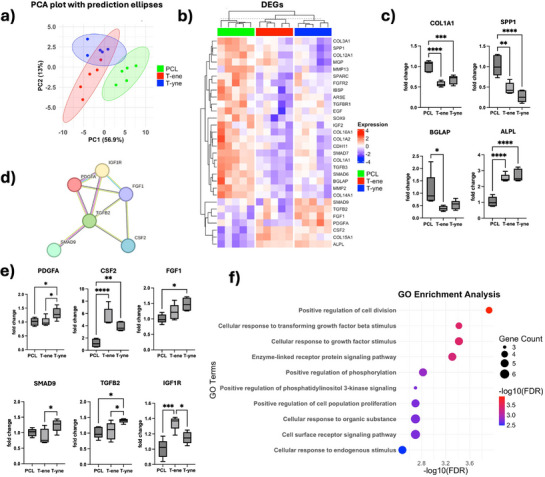
Results from the gene array for osteogenesis. (a) Principal component analysis (PCA) plot with prediction ellipses based on the significantly DEGs. (b) Significant DEGs visualized in a heatmap. (c) Graphs highlighting key osteogenic markers and their expression. (d) Displaying some of the upregulated genes in T‐ene group, which had (e) functional and physical protein associations, showed by the string network. (f) Enriched biological processes based on upregulated markers in the TATO‐thermosets. Data presented as mean with error bars representing SD, ^*^ < 0.05, ^**^ *p* < 0.01, ^***^ *p* < 0.001, ^****^ *p* < 0.0001. DEGs = differentially expressed genes.

Among the tested osteogenic markers, 30 markers were differentially expressed (Figure 5b). In total, 23 genes were significantly higher expressed in PCL compared to either T‐ene or T‐yne, while 7 genes were significantly higher expressed in T‐ene or T‐yne compared to PCL (Figure 5b; Figure S4c,d). In the corresponding PCA plot, PC1 accounts for 56.9% of the total variance, whereas PC2 accounts for 13% (Figure 5a). Key osteogenic markers, such as Collagen I alpha 1 (COL1A1), SPP1, and Osteocalcin (BGLAP), were all more highly expressed in PCL (Figure 5c). On the contrary, the early to mid‐phase osteogenic marker, ALPL, was significantly upregulated in T‐ene and T‐yne. The upregulated markers in the TATO‐group were further evaluated. Using the STRING database, 6 markers including CSF2, TGFB2, SMAD9, PDGFA, IGF1R, and FGF1 were identified for their protein‐protein interaction (Figure 5d–f). GO analysis predicted functional enrichment in Positive regulation of cell division, Cellular response to TGF‐β stimulus, and Cellular response to growth factor stimulus, among others (Figure 5f).

### 3.6 Comparable Osteogenic Differentiation of BMSC on T‐ene and T‐yne to PCL

**FIGURE 6:**
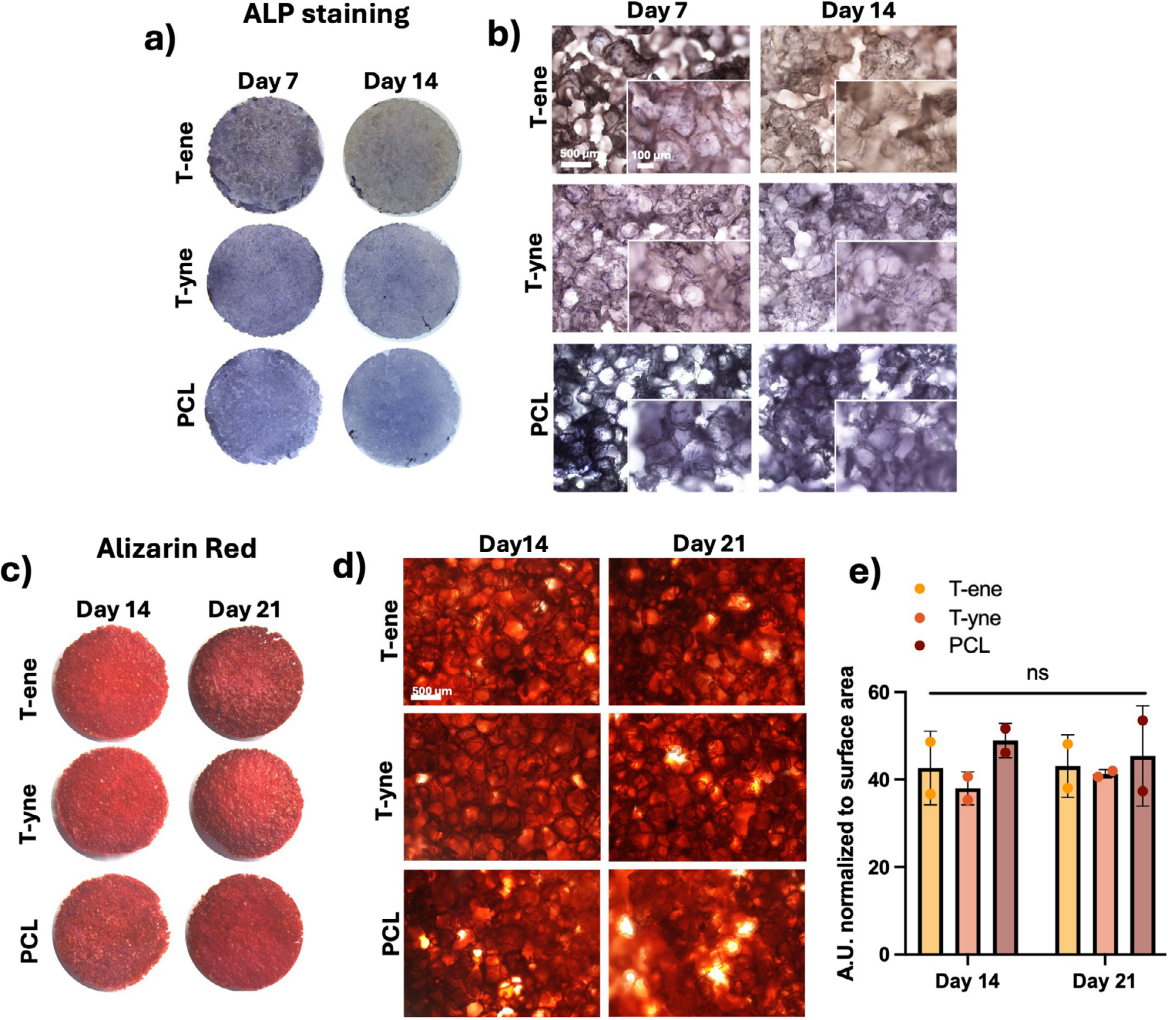
Macroscopic and microscopic images showing scaffolds after staining for (a,b) Alkaline Phosphatase Activity and (c,d) Alizarin Red S. (e) Quantitative data of Alizarin Red S staining adjusted for scaffolds surface area.

BMSC osteogenesis was functionally evaluated by their ALP activity and mineralization. Even though differences at the mRNA level were observed between the T‐ene, T‐yne and PCL groups, variations in their osteogenic functionality appeared to be minimal. Positive ALP staining was observed in all three groups, with color intensity appearing strongest on day 7, particularly for T‐ene and T‐yne (Figure 6a). Microscopically, no clear difference between the groups were observed (Figure 6b). Similarly, mineralization detected by Alizarin Red S staining developed over the time up to 21 days of osteogenic differentiation in all the groups (Figure 6c,d). The quantitative analysis revealed a slightly higher mineralization in the PCL group, but no statistical difference was detected (Figure 6e).

## 4 Discussion

Click chemistry is increasingly seen as a foundational tool for future regenerative materials, hailed as a groundbreaking discovery in modern chemistry. The overarching principles of click chemistry include chemo‐selectivity with no side‐reactions, solvent independent high fidelity with near‐complete conversion rates at equimolar ratios, and the production of virtually no byproducts [29]. Moreover, polymeric networks formed via click chemistry can be fully crosslinked under stoichiometric or off‐stoichiometric conditions, while simultaneously incorporating additional clickable functionalities [30]. Among clickable biomaterials, TATO‐based materials are highly scalable with low production costs and minimal batch‐to‐batch variation, making them attractive for translational applications [31]. Additionally, the monomers exhibit high chemical stability, enabling off‐the‐shelf availability and extended shelf life [17]. These characteristics suggest that TATO‐based materials may offer a cost‐effective and scalable alternative for future biomedical and tissue engineering applications.

For biomaterials to function effectively as scaffolding materials, formability, the ease with which a material can be shaped into a desired structure, is a critical property [32]. The TESTATO‐4PA, TESTATO‐4PTYA and TEMPIC monomers are viscous liquids at room temperature, enabling facile shaping at both room and physiological temperatures. On the contrary, the conventional synthetic polymers used in BTE are solid at room temperature and require either elevated temperatures, often exceeding physiologically acceptable ranges, or dissolution in hazardous organic solvents to enable processing and shaping [33, 34]. For example, the TATO‐based materials may be easily used for coating or casting without heat or solvent while coating with polycaprolactone (PCL) requires heating the polymer and/or dissolving it in solvent (e.g., acetone, chloroform, methylene chloride, and formic acid), which presents significant health and safety risks, necessitating stringent precautions during use  [34, 35]. PCL and other synthetic polymers could also be used to design highly porous scaffolds, either with pressure or temperature in combination with a foaming agent. However, to use the same polymers to fabricate porous foams that can be directly injected and cured in situ, still represents a challenge [36, 37]. The excellent formability of the TATO‐based materials was proven through the fabrication of highly porous scaffolds made by a salt‐leaching technique, extrusion‐based 3D printing, and selective laser sintering [20, 38]. Additionally, the attractive property of processing and shaping at room or physiological temperatures may enable in situ tissue‐engineered medicine at the surgical site, potentially enhancing intraoperative usability and clinical translation.

Biocompatibility is a fundamental requirement for biomaterials. The classical definition states that a biomaterial should possess the ability “to perform with an appropriate host response in a specific situation” [39]. While broad, this definition has been refined in specific contexts such as tissue engineering. One proposed paradigm defines biocompatibility as the ability of a scaffold or matrix to perform “without eliciting any undesirable local or systemic responses in the eventual host” [40]. Several TATO systems have been investigated, and previous studies have reported no signs of cytotoxicity, genotoxicity, inflammation, or skin irritations [18, 19, 20, 41]. We also confirmed that fully HEV light‐cured T‐ene and T‐yne did not exhibit any cytotoxicity [20]. Nonetheless, curing efficiency needs careful optimization depending on conditions such as the intensity and source of the curing light and exposure time as well as the size and design of the targeted scaffolds. Notably, we found that the T‐yne required more energy input and longer exposure time to achieve complete polymerization due to the TYC reaction allowing for each alkyne bond to couple with two thiol bonds. This resulted in a stiffer polymeric network. T‐ene exhibited higher crosslinking efficiency, and the resulting polymer network was more elastic and flexible [14]. Suboptimal curing methods as well as insufficient washing may lead to the presence of residual unreacted monomers, causing initial yet transient adverse effects on cell viability and functions. While we did not observe functional changes, the RNAseq analysis suggested altered immunomodulation and inflammatory responses in BMSC on T‐yne. Additionally, unlike T‐ene, extracts from freshly cured T‐yne with HEV light caused cell death and reduced growth, likely due to unreacted monomers leached into the medium (Figure S5). However, in the present study, the salt‐leached scaffolds made of T‐ene and T‐yne did not show signs of cytotoxicity nor other adverse effects. This was consistent across multiple assays, including the ex ovo CAM assay or in vitro studies assessing adhesion and osteogenic differentiation, demonstrating equivalent biocompatibility to PCL once fully cured. Nevertheless, despite the stable chemistry of the T‐ene and T‐yne thermosets once fully cured, there remains a potential risk of residual monomers, crosslinkers, or reactive oxygen species being trapped within the construct, which could negatively affect biocompatibility. Therefore, adequate light curing is essential, and when fabrication macro‐scale structures, careful consideration of construct size and design is crucial to ensure complete conversion of all reactive monomers and crosslinking throughout the material. However, the monomer conversion for the T‐ene and T‐yne is near to completion upon exposure to light due to the step‐growth polymerization. This can be compared to the acrylic systems, that due to the vitrification phenomena have unreacted monomers present even after the materials has been crosslinked [14, 18, 42].

Given the difference in curing efficiency, T‐yne would be more suitable for applications where the material is prepared in a laboratory setting and can undergo a post‐processing washing step. In contrast, T‐ene is better suited for chair‐side preparation or direct application at the surgical site, making it more favorable for high‐efficiency and rapid‐curing applications. T‐ene may be particularly advantageous in scenarios requiring the curing of larger material volumes or where rapid polymerization is essential, for instance, in 3D printing, where faster crosslinking improves process efficiency and geometrical accuracy. Furthermore, due to its favourable rheological properties and biocompatibility, co‐printing T‐ene scaffolds alongside cell‐laden bioinks holds strong potential to advance the biofabrication of multiphasic, structurally complex bone implants.

The upregulated genes in T‐ene and T‐yne were found to be functionally associated with cell division and growth factor signaling. An inverse relationship was observed between proliferation and osteogenic commitment in BMSCs, where high proliferative activity during the early differentiation phase progressively declined as the cells became committed to the osteogenic lineage [43]. For example, transforming growth factor β 2 (TGF‐β2), which was upregulated in T‐yne, is one of three TGF‐β isoforms known to promote MSC proliferation in vitro [44]. As part of the TGF‐β2 signaling pathway, TGF‐β2 supports early lineage commitment and differentiation of pre‐osteoblasts, suggesting that BMSC seeded on T‐yne were in a more proliferative and less differentiated state [45, 46]. Fibroblast growth factor‐1 (FGF1), which was significantly upregulated in T‐yne and moderately increased in T‐ene, also plays a key role in MSC proliferation [47]. Similarly, Platelet‐derived growth factor subunit A (PDGFA), which promotes both MSC proliferation and migration, was significantly elevated in T‐yne [48]. In T‐ene, Insulin‐like growth factor‐1 receptor (IGF1R) was significantly upregulated, with a similar trend observed in T‐yne relative to PCL. Activation of the IGF1 signaling pathway, initiated upon ligand binding to IGF1R, regulates multiple processes, including MSC proliferation, differentiation, and the promotion of pre‐osteoblast maturation [49]. Assessment of the osteogenic potential of T‐ene, T‐yne, and PCL revealed modest differences at the gene expression level. The early osteogenic marker alkaline phosphatase (ALPL) was significantly upregulated in both T‐ene and T‐yne compared to PCL [50]. In contrast, osteocalcin (BGLAP) and osteopontin (SPP1), which are well established late‐stage osteogenic markers, were upregulated in PCL at day 14, based on the osteogenesis gene array analysis [51, 52]. Interestingly, alpha‐1 type I collagen (COL1A1), an early osteogenic marker and encoding a major part of the collagen I triple helix, was also more highly expressed in PCL samples [53]. Despite the early marker COL1A1 being elevated in PCL, the simultaneous upregulation of BGLAP and SPP1 may suggest that the osteogenic differentiation was more advanced in the PCL group at the day 14 timepoint. However, since gene expression was only assessed at a single timepoint, the temporal dynamics of osteogenesis across the different materials remain unclear. Additional timepoints would be necessary to determine the progression and timing of gene expression changes. Minor differences in porosity and surface area among the scaffolds (Figure 1; Figures S1 and S2) could potentially affect the osteogenic gene expression. Interestingly, the functional assays, Alkaline Phosphatase (ALP) activity and Alizarin Red S staining, did not reflect the differences observed at a transcriptomic level. This discrepancy may be due to the normalization of Alizarin Red S absorbance values to scaffold surface area. ALP staining showed only a slight decrease in color intensity, and quantitative analysis of Alizarin Red staining revealed minimal differences between days 14 and 21, potentially indicating that mineral deposition had reached saturation across all groups.

In the present study, BMSC behaviors on TATO‐based biomaterials were evaluated, revealing comparable functional properties and osteogenic differentiation capacity despite differences in gene expression. To tailor the materials for BTE, further functionalization may be preferred to add and/or improve osteoconductivity and osteoinductivity. TATO monomers contain multiple reactive sites such as alkene or alkyne groups, which provide accessible points for chemical modification. These can be used to covalently attach bioactive molecules (e.g., peptides, growth factors) to improve bioactivity of the materials. Additionally, the hydrophobic and low‐viscosity nature of uncured TATO formulations makes them compatible with a broad range of organic and inorganic additives, such as hydroxyapatite, silica nanoparticles, or drug‐loaded microspheres, allowing for homogeneous mixing and co‐curing, thereby broadening their applicability across diverse biomedical contexts [14]. Future work will focus on optimizing functionalization strategies and evaluating the controlled release of therapeutic agents from TATO‐based composites as well as in vivo long‐term validation, paving the way for their use in bone regeneration therapies.

## 5 Conclusion

In this study, two novel TATO‐crosslinked thermosets were evaluated comprehensively as a new class of clickable, formable, and biocompatible scaffolds. While T‐ene and T‐yne demonstrated comparable cytocompatibility and osteogenic functionality to PCL, differences in curing kinetics and gene expression profiles suggested application‐specific advantages, with T‐ene preferred for rapid, clinically accessible settings and T‐yne better suited to laboratory‐controlled fabrication requiring enhanced rigidity. TATO thermosets offer the advantage of being manufactured at room or physiological temperatures without the use of harmful solvents, in contrast to conventional thermopolymers like PCL. Furthermore, the abundant reactive sites and excellent compatibility with a variety of bioactive and structural additives position TATO‐based materials as versatile systems for advanced biofabrication and tissue regeneration. For BTE, future investigations will be required to enhance their osteoconductive and osteoinductive properties through chemical functionalization and additive incorporation, alongside in vivo studies to validate their long‐term safety, degradability, and regenerative potential in clinically relevant models.

## Author Contributions

Å.J. contributed to writing, original draft, writing, review and editing, conceptualization, data curation, formal analysis, investigation, and visualization. S.Y. contributed to writing, original draft, writing, review and editing, conceptualization, data curation, formal analysis, investigation, and supervision. D.J.H. contributed to writing, review and editing, conceptualization, resources, and funding acquisition. M.Y. contributed to writing, review, and editing, data curation, and supervision. S.M‐A. contributed to writing, review, and editing, and resources. C.G. contributed to writing, review and editing, supervision, and funding acquisition. M.M. contributed to writing, review and editing, conceptualization, resources, and funding acquisition. K.M. contributed to writing, review and editing, conceptualization, resources, project administration, supervision, and funding acquisition.

## Conflicts of Interest

The authors declare no conflicts of interest.

## Supporting information




**Supporting File**: adhm70507‐sup‐0001‐SuppMat.docx.

## Data Availability

The data that support the findings of this study are available from the corresponding author upon reasonable request.
