# Supplementary material for: Triazine‐Trione Thermosets with High Processability for Scaffold Applications in Bone Tissue Engineering
Source: Adv Healthc Mater. 2025 Nov 22;15(7):e04163. doi: 10.1002/adhm.202504163 (PMC12908209; doi:10.1002/adhm.202504163)
Supplement: Supplementary file 1 — Supporting File: adhm70507‐sup‐0001‐SuppMat.docx. [file ADHM-15-0-s001.docx]

**Supporting information**

**Supplementary materials and methods**

**Compression test**

TATO materials were shaped into discs with a diameter of 9 mm and a height of 2 mm using a Teflon mold. Compressive behavior was tested using an Instron 5966 universal testing machine (Instron, USA) equipped with a 10 kN load cell and a compression rate of 1 mm/min. In the graph, up to 60 % strain is visualized, but the testing was terminated upon reaching a maximum load of 9000 N. Bluehill software was used for data acquisition and analysis. For T-ene, the elastic modulus was calculated in the compressive strain range 0-25 %, as this range was most relevant for comparison with T-yne (n = 5).

**Cytotoxicity testing: Test of extract**

To make the extracts, 3 mL complete medium were added to wells coated with 2 g of TATO-2 and TATO-3 in a 6 well plate. For PCL, 2 g of granules were added to the well. The extracts were incubated for 24 h in a 5% CO_2_ humidified incubator. Control medium was also incubated.

In a 96-well plate, BMSC cells were seeded, with the concentrations of 2,000 cells/well. The next day, the medium was removed, and the extracts were added, 100 µL/well. 2 drops/mL of green dye (NucGreen™ Dead 488: R37109; Invitrogen, US) to stain dead cells were added. Well plates were incubated in Incucyte® S3 Live-Cell Analysis System (Sartorius, Germany) for 48 h.

**Supplementary results**

**Decrease in BMSC proliferation and apoptosis after exposure to TATO-3 extract**

After 48 hours of incubation with extract, the BMSC had proliferated, and the cell confluency had increased from between 20-25 % to over 50% for the control, TATO-2 and PCL. However, for TATO-3, the cell confluency had decreased to around 15 %. There was also an increase in the number of cells stained green in the TATO-3 group, meaning an increase in dead cells. The number of green dyed cells were stable for the other groups (Fig. S4a-c).


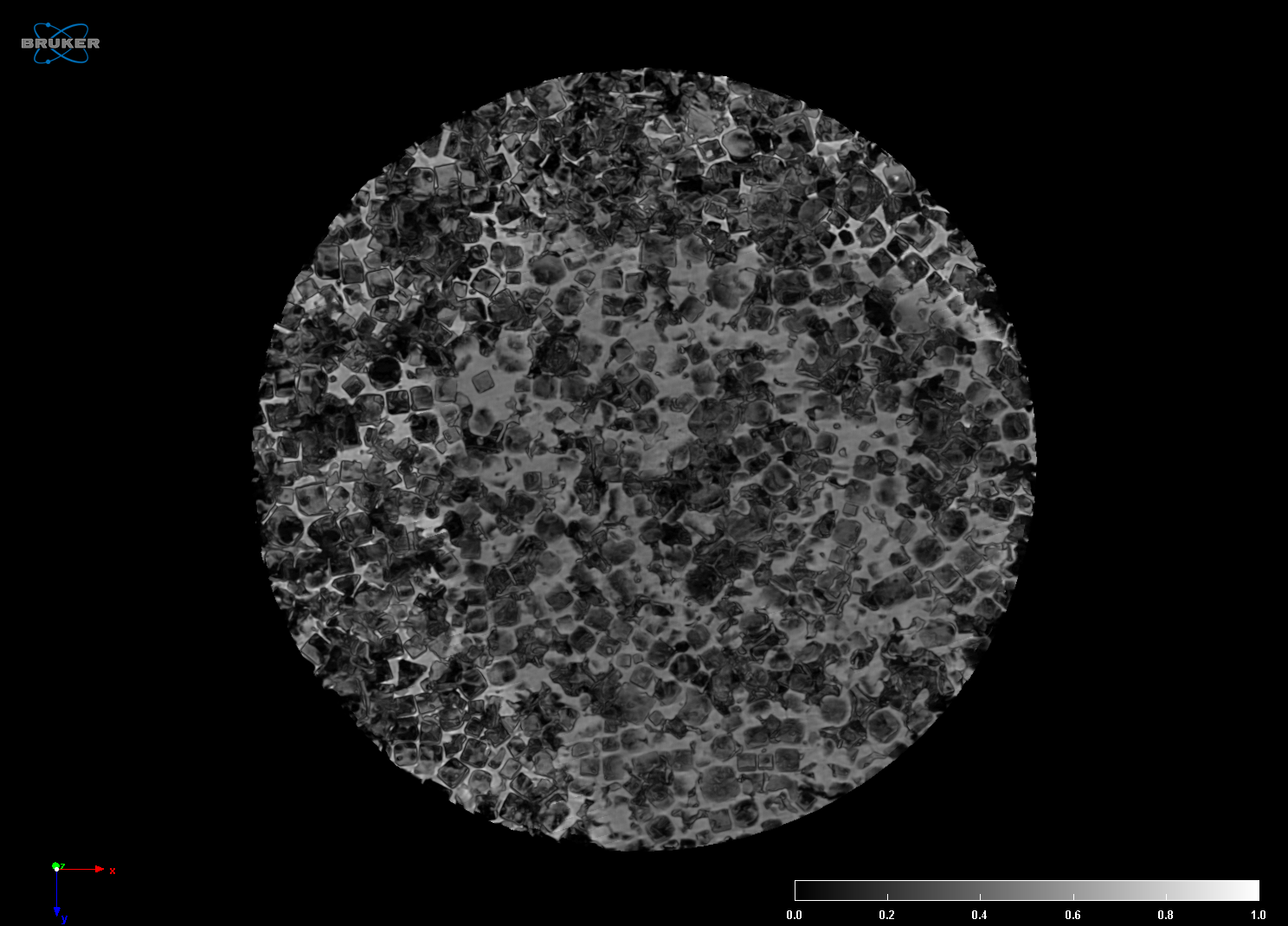


**Figure S1.** micro-CT image of PCL, with a porosity of 88,11 % (± 0.178), n = 3.

| **Object surface / volume ratio** | | |
| --- | --- | --- |
| **Material** | **1/μm** | **±SD** |
| T-ene | 0,05251364 | 0,00605618 |
| T-yne | 0,04425577 | 0,00605919 |
| PCL | 0,07464825 | 0,00180448 |

**Figure S2.** Object surface / volume ratio obtained from micro-CT analysis of porous scaffolds


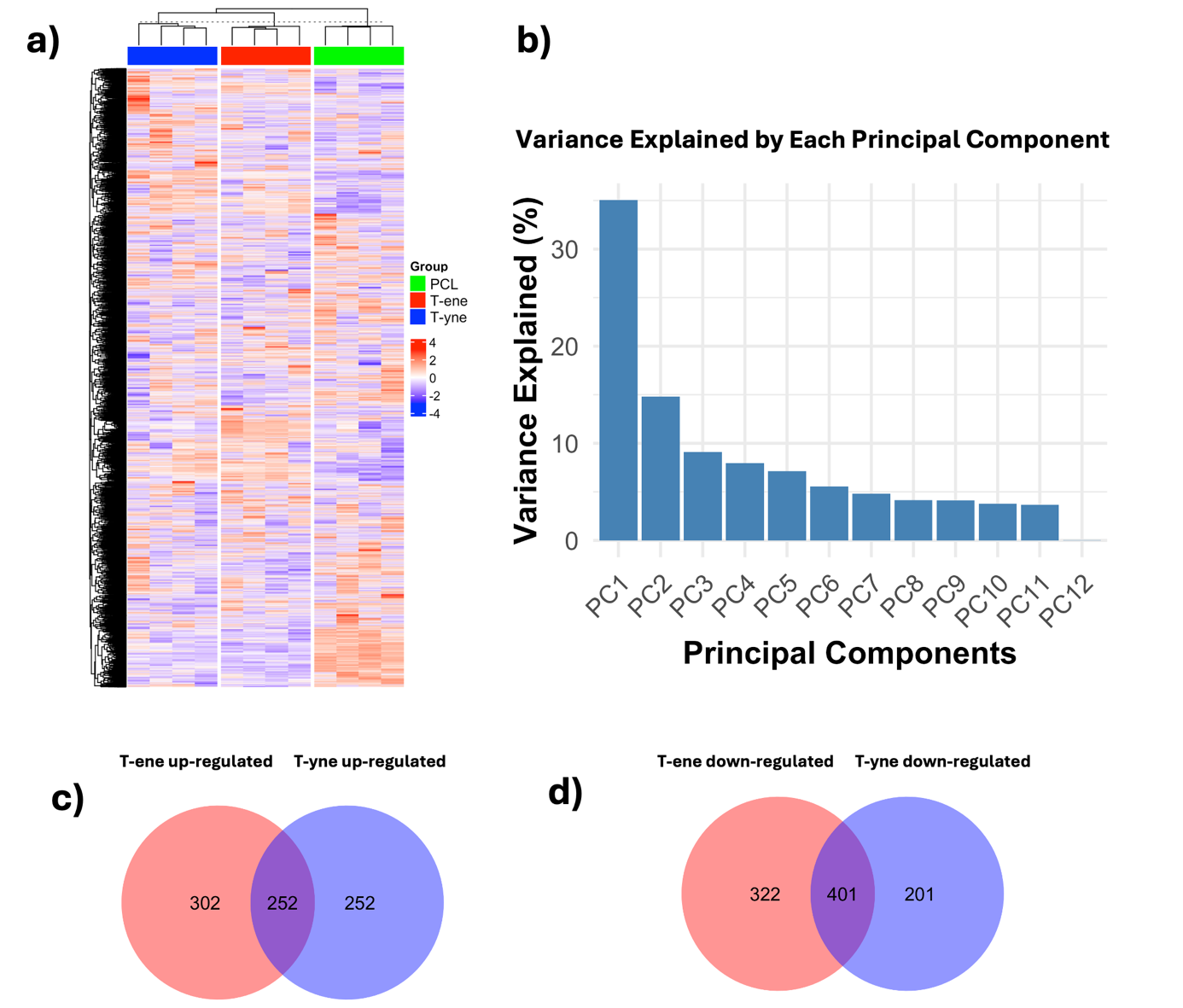


**Figure S3.** Heatmap showing all genes except genes with missing values and low-variance genes (variance < 0.1). Z-score normalization is performed, and hierarchical clustering is done using Euclidean distance and complete linkage. b) Graph showing the Variance explained by each Principal Component, related to the PCA plot in Fig. 2a. d-e) c-d) Venn diagrams showing the number of upregulated and downregulated genes of T-ene and T-yne compared to PCL.


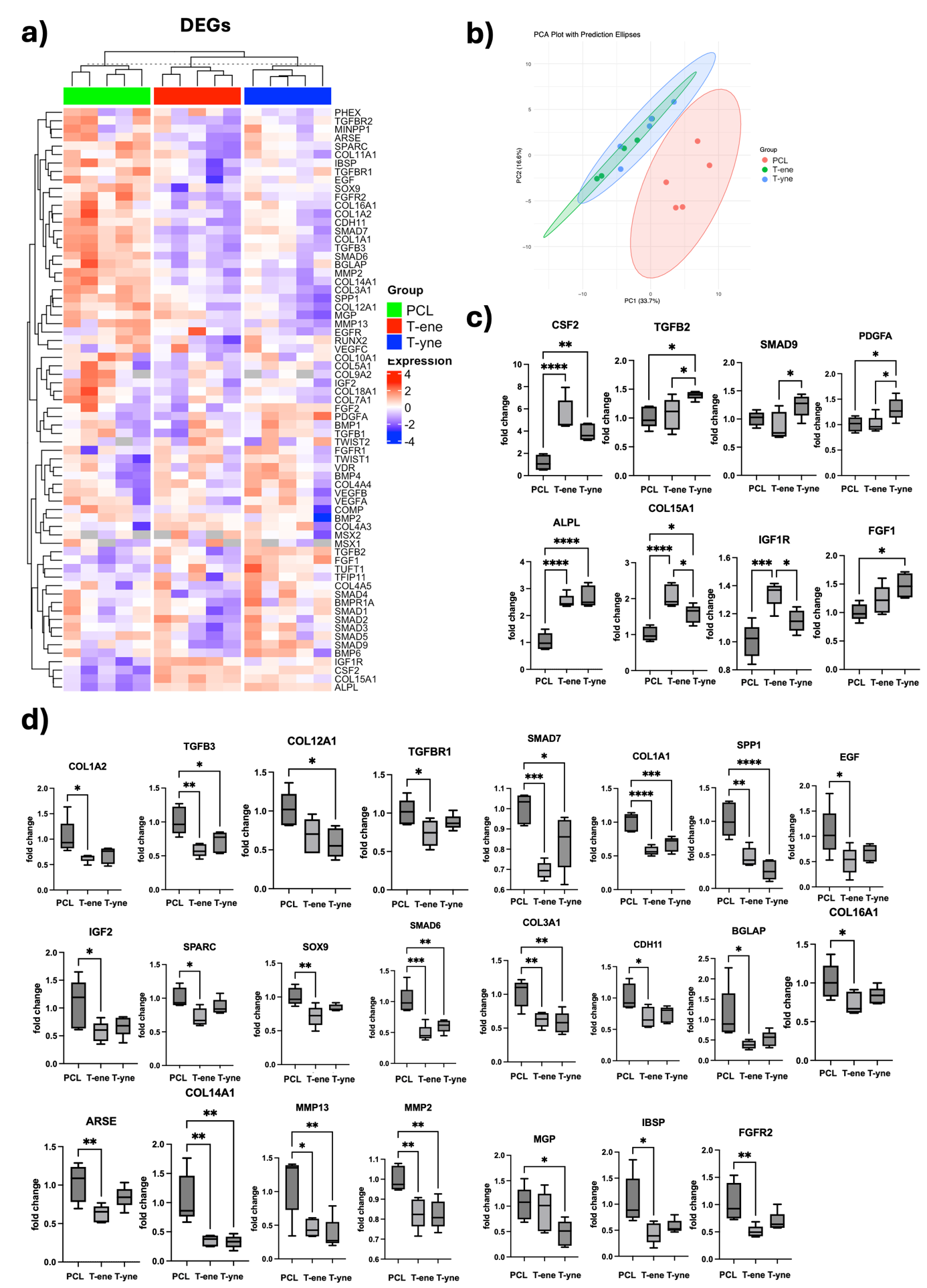


**Figure S4.** a) Heatmap showing all the included genes from the gene array for osteogenesis and the b) PCA plot with prediction ellipses. c) All the individual genes where T-ene or T-yne were significantly up-regulated compared to PCL, and d) all the individual genes where PCL were significantly up-regulated compared to T-ene and T-yne. *p < 0.05, ** p < 0.01, ***p < 0.001, ****p <0.0001.


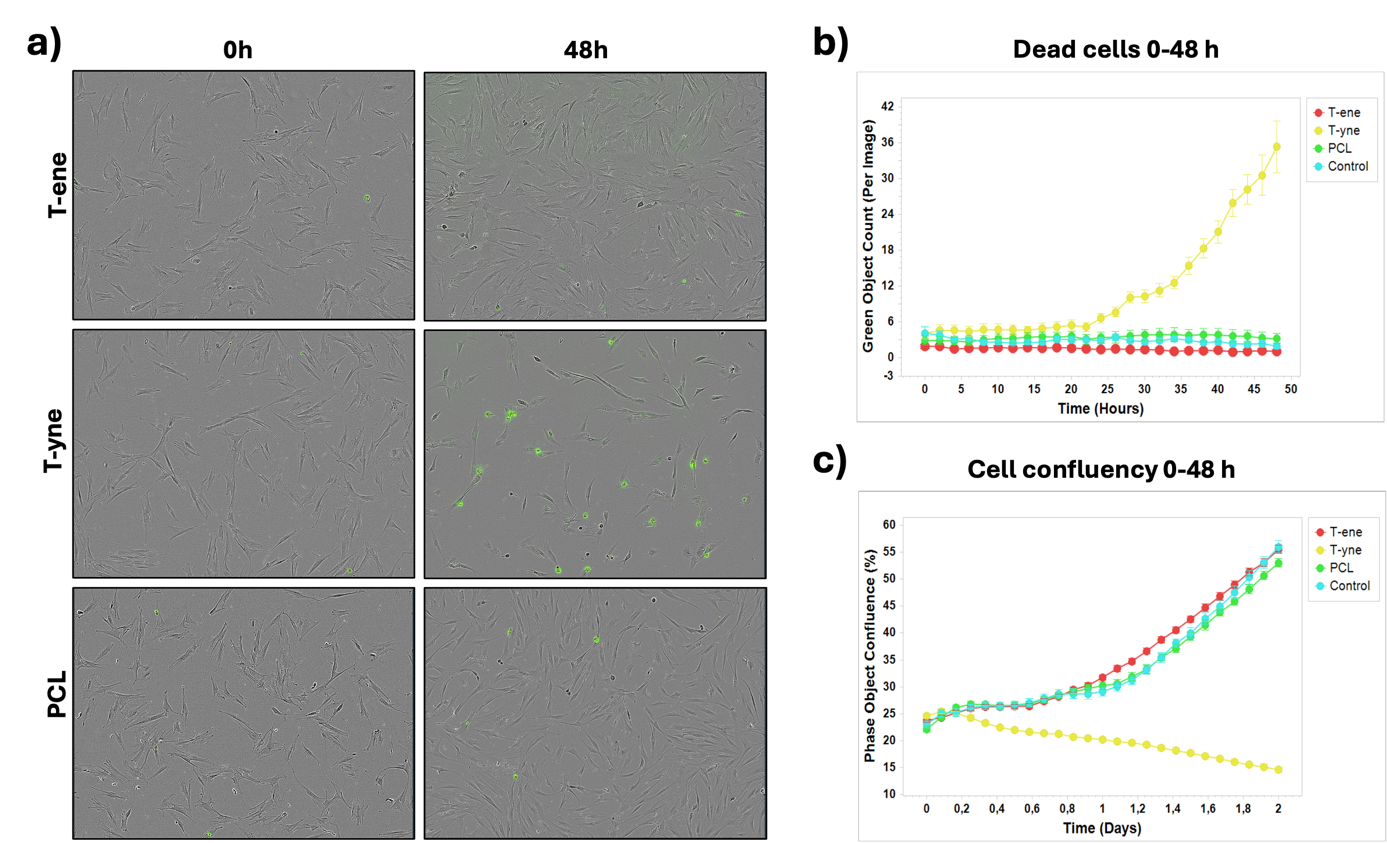


**Figure S5.** BMSC response to extract showing a) cell morphology and confluency after 0 hours and 48 hours, b) number of cells stained green and the c) development of cell confluency over time during incubation with the extract.
